# Plasma Lipoprotein-Associated Phospholipase A_2_ Levels Correlated with the Cardio-Ankle Vascular Index in Long-Term Type 2 Diabetes Mellitus Patients

**DOI:** 10.3390/ijms17050634

**Published:** 2016-04-27

**Authors:** Kazuhiko Kotani

**Affiliations:** 1 Division of Community and Family Medicine, Jichi Medical University, 3311-1 Yakushiji, Shimotsuke-City, Tochigi 329-0498, Japan; kazukotani@jichi.ac.jp; Tel.: +81-285-58-7394; Fax: +81-285-44-0628; 2 Department of Clinical Laboratory Medicine, Jichi Medical University, 3311-1 Yakushiji, Shimotsuke-City, Tochigi 329-0498, Japan

**Keywords:** arterial stiffness, atherosclerosis, CAVI, disease duration, platelet-activating factor acetylhydrolase, Lp-PLA_2_

## Abstract

The circulating levels of lipoprotein-associated phospholipase A_2_ (Lp-PLA_2_) can be a simple, but practical and useful marker of cardiovascular disease (CVD). As limited studies are available in patients with diabetes mellitus (DM), further studies are needed to establish the clinical application of Lp-PLA_2_ in DM practice. The present study investigated the correlation between Lp-PLA_2_ and the cardio-ankle vascular index (CAVI), a recent marker of arterial stiffness, in DM patients according to their diabetes duration. Clinical data, including the plasma Lp-PLA_2_ mass and CAVI values, were collected from CVD-free type 2 DM female patients (*n* = 65, mean age 62 years, mean hemoglobin A1c 7.0%). The Lp-PLA_2_ level of patients with a diabetes duration of <10 years (*n* = 40:20.2 IU/mL) was not significantly different from that of patients with a diabetes duration of ≥10 years (*n* = 25:20.5 IU/mL), while the CAVI level was significantly higher in patients with ≥10 years (9.0) than in those with <10 years (8.1; *p* < 0.05). A stepwise multiple regression analysis found a positive correlation between the Lp-PLA_2_ and CAVI levels (β = 0.43, *p* < 0.01) in patients with a diabetes duration of ≥10 years. This correlation between Lp-PLA_2_ and CVAI suggests the possible use of Lp-PLA_2_ in DM patients with long-term disease. Further studies on Lp-PLA_2_ are warranted in DM practice in relation to the disease duration.

## 1. Introduction

Hyperglycemia causes endothelial dysfunction, vascular inflammation, arterial wall hypertrophy and fibrosis, which are all interrelated processes that lead to vascular damage with arterial stiffness [1,2]. Cardiovascular disease (CVD) is, therefore, the major cause of morbidity and mortality in diabetes mellitus (DM) patients [1,2]. For regulation, blood markers for CVD risks in DM patients remain to be explored.

Lipoprotein-associated phospholipase A_2_ (Lp-PLA_2_; formerly known as platelet-activating factor acetylhydrolase) is a member of the intracellular and secretory phospholipase enzyme family secreted by activated macrophages [3,4]. While Lp-PLA_2_ hydrolyzes oxidized phospholipids (this is thought to be atheroprotective), it can produce oxidized fatty acids and lysophosphatidylcholine, which can trigger inflammation (leading to atherosclerosis) [3,4]. A study using a meta-analysis [5] has reported that an increase of Lp-PLA_2_ mass/activity can predict the development of CVD (even though there are some differences in the prediction ability of CVD between the Lp-PLA_2_ activity and mass [6,7]). The guidelines of several societies advocate the use of Lp-PLA_2_ when evaluating CVD risks [8,9]; however, the results of Mendelian randomization and phase III randomized control trials on inhibitors of Lp-PLA_2_ do not always support the causal involvement of Lp-PLA_2_ in the development of CVD [10]. Thus, Lp-PLA_2_ is currently regarded as a simple (non-causal), but practical marker for CVD [5,10], and we must further consider how to use Lp-PLA_2_ in the clinical setting.

Limited studies regarding Lp-PLA_2_ in DM practice have so far been conducted. The increase of Lp-PLA_2_ activity is reported to predict the development of CVD in type 2 DM patients [6,7]. On the other hand, an additional review indicates no clear association of Lp-PLA_2_ levels with type 2 DM [11]. Multi-faceted studies remain necessary to establish the clinical application of Lp-PLA_2_ to DM patients.

Arterial stiffness is a surrogate marker of CVD, and it is typically assessed by pulse wave velocity (PWV) in the clinical setting [1,2]. A major problem in the use of PWV for determining the extent of arterial stiffness is that it is significantly affected by blood pressure (BP) during the measurement [12,13,14,15,16,17]. The cardio-ankle vascular index (CAVI) is a recently-developed alternative method for assessing arterial stiffness and is independent of BP during the measurement, thus offering an advantage over PWV and accumulating evidence in vascular research areas [12,13,14,15,16,17].

In a few studies, the results of the association between Lp-PLA_2_ and arterial stiffness are discrepant [18,19]. To date, there have been no studies that examine the association of Lp-PLA_2_ with the CAVI. In the present study, the correlation between circulating Lp-PLA_2_ mass and the CAVI levels was investigated in type 2 DM patients. Given that the diabetes duration (e.g., <10 or ≥10 years) is a clinical determinant of the influence on the DM-related pathology as shown previously [20,21,22,23,24], we performed the study according to the disease duration.

## 2. Methods

A total of sixty-five type 2 DM female patients were enrolled in this cross-sectional study. By the inclusion criteria, all patients were non-smokers, non-alcohol abusers and were medication and/or supplementation free, except for hypoglycemic therapies (*i.e.*, special diets, oral agents, insulin), lipid-lowering therapies and anti-hypertensive therapies. The exclusion criteria were that all patients had to be free of any acute infectious diseases and had to have no prior history of cardiovascular, thyroid, rheumatologic or severe liver disease. The protocol was approved by the ethics committee of human research at Jichi Medical University, and all patients gave informed written consent.

The clinical data from each patient were obtained after an overnight fast. Body mass index (BMI) was calculated based on the weight and height measured, while lightly dressed and without shoes. Systolic and diastolic BP was measured in the patient’s right arm with a mercury sphygmomanometer, while the patient was in the seated position. Then, the mean BP was calculated using the following equation: diastolic BP plus (systolic BP minus diastolic BP)/3. Serum total cholesterol (T-Chol) was enzymatically measured. Hemoglobin A1c (HbA1c) was measured by a high-performance liquid chromatography. The plasma Lp-PLA_2_ mass level was measured by an enzyme-linked immunosorbent assay (Cusabio Biotech Co., Ltd., Wuhan, China), with 8.0% and 8.5% of intra- and inter-coefficient of variations.

Obesity was defined as having a BMI of ≥25 kg/m^2^ [25]. The presence of nephropathy was determined in cases with >300 mg/g creatinine levels by the urinary albumin/creatinine ratio as measured with the immunoassays [26]. The presence of retinopathy was determined by ophthalmologists using ophthalmoscopic examinations [27]. The CAVI was determined by using the VaSera system (Fukuda Denshi Co., Ltd., Tokyo, Japan). All patients were in a supine position on a bed for 10 min before the CAVI measurement. This measurement requires the placement of electrodes for the electrocardiogram on the wrists, a microphone for phonocardiogram on the sternum and BP cuffs around the extremities. The upper arm and ankle pulse waves, as well as the BP, are then measured. The CAVI value is calculated based on the PWV from the aortic valve origin to the ankle region and BP at the upper arm [12,13,14,15,16,17]. The formula for the value (as a non-unit quantity), driven using the Bramwell–Hill equation and the stiffness parameter β, is as follows: *ln*(*Ps*/*Pd*) *×* 2ρ/Δ*P × PWV*^2^ (ρ: blood density, *Ps*: SBP, *Pd*: DBP, Δ*P*: *Ps*–*Pd*, *PWV* between the aortic and the ankle value) [11,12,13,14].

The data are expressed as the means ± standard deviations, the medians (interquartile ranges) or patient numbers (%). The between-group differences were analyzed using *t*-tests or χ^2^-tests. Simple linear correlation tests (Pearson tests) were used to observe the correlations between variables. Subsequently, stepwise multiple linear regression analyses were used to identify variables correlated with CAVI (an outcome variable). The Lp-PLA_2_ values were log-transformed in the analyses because of their skewed distributions. Statistical significance was defined as a *p*-value <0.05.

## 3. Results

Table 1 shows the clinical characteristics of the study patients stratified by a diabetes duration of <10 or ≥10 years. Patients with a diabetes duration of ≥10 years (*n* = 25) showed a significantly longer diabetes history, as well as a significantly higher prevalence of lipid-lowering therapies, hypoglycemic therapies, nephropathy and retinopathy than those with a duration of <10 years (*n* = 40). Patients with a diabetes duration of ≥10 years had a significantly lower T-Chol level than those with a duration of <10 years. The Lp-PLA_2_ level of patients with a diabetes duration of ≥10 years did not significantly differ from that of patients with a duration of <10 years, but the CAVI level was significantly higher in patients with a diabetes duration of ≥10 years than in those with a duration of <10 years.

Table 2 shows the findings regarding the correlations between the patients’ characteristics and CAVI levels. Simple correlation and stepwise multiple regression analyses showed a significant positive correlation between age and CAVI in patients with a diabetes duration of <10 years. In patients with a diabetes duration of ≥10 years, there was a significant positive correlation between age and CAVI as well as between the disease duration and CAVI in simple correlation tests. We detected a tendency toward a positive correlation between Lp-PLA_2_ and CAVI levels. A subsequent stepwise multiple regression analysis revealed that the CAVI level was significantly-, independently- and positively-correlated with Lp-PLA_2_, along with the diabetes duration (a significant positive correlation) and obesity (a significant inverse correlation).

## 4. Discussion

The present study identified a significant positive correlation between the circulating Lp-PLA_2_ mass and CAVI levels in type 2 DM female patients with a diabetes duration of ≥10 years. Taking the presumed nature of these two markers for CVD into consideration, the finding of a positive correlation between Lp-PLA_2_ (which is simply, but positively related to CVD [5]) and the CAVI (which is related to arterial stiffness as a surrogate for CVD [12,13,14,15,16,17]) may partly reflect the increase in the CVD burden in patients with long-term disease [20,21,22]. This finding suggests the possible use of Lp-PLA_2_ measurement for assessing atherosclerotic pathophysiology in long-term DM patients (the use is restricted, but can be effective in the target population).

The positive correlation between Lp-PLA_2_ and CAVI also appears to be consistent with an earlier study showing a significant positive association between Lp-PLA_2_ and various arterial function test results in patients with coronary disease [18]. As for the conflicting results regarding the Lp-PLA_2_-arterial stiffness correlation across existing studies (including our present study) [18,19], the differences in the studied populations, methods of assessing arterial stiffness and the adjusted variables used in the statistical analyses might be related to the different results. The use of the CAVI is one aspect in which the present study differs from the earlier studies [18,19]. Unlike PWV, the CAVI is independent of BP, which may be a confounding factor when attempting to observe an association with the development of CVD [11,12,13,14]. Given the merit of the CAVI measurement [12,13,14,15,16,17], it would be valuable to note that the correlation of Lp-PLA_2_ with arterial stiffness was confirmed using the CAVI.

The mechanisms underlying our present findings regarding the Lp-PLA_2_-CAVI correlation in long-term DM patients remains unclear. A long duration has been reported to be required to manifest the development of CVD-related outcomes in DM patients [20,21,22]. The need for a long-term period of time to elapse before apparent vascular manifestations via the diabetic state (hyperglycemia and its related glycation products [1,2]) and Lp-PLA_2_ [3,4,5] may be related to the findings from our present study.

The positive correlation between age and CAVI observed in the present study seems to be natural [15]. The findings of the higher CAVI level in patients with ≥10 years than in those with <10 years and the positive correlation between the disease duration and CAVI in patients with ≥10 years also seem to support the previous study showing the positive effect of diabetes duration on the development of CVD [20,21,22]. The inverse correlation between obesity and CAVI was found in the present study. The inverse correlation has been seen in previous studies, even though the correlation may not always be significant [28,29,30]. The fact that there is a trend for BMI to decline among aged and long-term DM patients [31] may partly affect their inverse correlation. The explanation for it should be further investigated.

The present study is associated with some limitations. First, the sample size was relatively small. Second, given the presence of gender difference in the CAVI values [15], we strictly limited the analysis to female patients only. Third, while we used a 10-year duration as a long-term period as shown previously [20,21,22,23,24], the duration may be shortened or lengthened to allow for confirmation of the relationship between Lp-PLA_2_ and CAVI levels. Studies with a larger sample size, enrolling male patients and different divisions for diabetes durations are planned in the future. Finally, this study had a cross-sectional design, and the CVD outcomes were not followed up. These must be also addressed in future work.

## 5. Conclusions

In summary, the present study revealed a positive correlation between the plasma Lp-PLA_2_ mass and CAVI levels in patients with a diabetes duration of ≥10 years. This correlation between Lp-PLA_2_ and CVAI suggests the possible use of Lp-PLA_2_ in DM patients with long-term disease. The findings are preliminary, but will facilitate more studies regarding the clinical application of Lp-PLA_2_ in DM practice in relation to the disease duration.

## Figures and Tables

**Table 1:** Clinical data of the patients according to the diabetes duration.

Parameters	All (*n* = 65)	<10 Years (*n* = 40)	≥10 Years (*n* = 25)	*p*
Age (years)	62 ± 9	61 ± 9	63 ± 8	0.24
Diabetes duration (years)	8.6 ± 7.6	3.3 ± 3.1	16.5 ± 5.0	<0.01 **
Obesity (presence)	31 (48%)	20 (50%)	11 (44%)	0.64
Hypertension (presence)	40 (62%)	21 (53%)	19 (76%)	0.06
Mean blood pressure (mmHg)	96 ± 11	95 ± 9	98 ± 13	0.25
Anti-hypertensive drugs (use)	32 (49%)	16 (40%)	16 (64%)	0.06
Hypercholesterolemia (presence)	52 (80%)	32 (80%)	20 (80%)	0.99
Total cholesterol (mg/dL)	196 ± 33	205 ± 32	182 ± 30	<0.01 **
Lipid-lowering drugs (use)	34 (52%)	17 (43%)	17 (68%)	0.045 *
Hemoglobin A1c (%)	7.0 ± 1.3	6.8 ± 1.4	7.4 ± 1.1	0.06
Hypoglycemic therapies (use)	39 (60%)	16 (40%)	23 (92%)	<0.01 **
Diabetic nephropathy (presence)	21 (32%)	9 (23%)	12 (48%)	0.03 *
Diabetic retinopathy (presence)	23 (32%)	3 (8%)	12 (48%)	0.01 **
Lp-PLA_2_ (IU/mL)	20.5 (13.3–26.1)	20.2 (11.9–27.0)	20.5 (14.8–25.1)	0.96
CAVI	8.5 ± 1.2	8.1 ± 1.1	9.0 ± 1.1	<0.01 **

Lp-PLA_2_: lipoprotein-associated phospholipase A_2_; CAVI: cardio-ankle vascular index. The data are presented as the means ± standard deviations, medians (interquartile ranges) or the numbers (%). Significance level (between patients with <10 and ≥10 years): * *p* < 0.05, ** *p* < 0.01.

**Table 2:** Correlations with the CAVI levels according to the diabetes duration.

Parameters	<10 Years		≥10 Years	
	*r* (*p*)	β (*p*)	*r* (*p*)	β (*p*)
Age (years)	0.55 (<0.01 **)	0.48 (<0.01 **)	0.50 (0.01 *)	NE
Diabetes duration (years)	0.10 (0.55)	NE	0.56 (<0.01 **)	0.50 (<0.01 **)
Obesity (presence)	−0.31 (0.06)	−0.27 (0.07)	−0.34 (0.09)	−0.36 (0.02 *)
Hypertension (presence)	0.01 (0.94)	NE	0.10 (0.65)	NE
Hypercholesterolemia (presence)	0.12 (0.46)	0.18 (0.20)	0.34 (0.10)	0.17 (0.27)
Hemoglobin A1c (%)	−0.12 (0.46)	NE	−0.10 (0.62)	NE
Diabetic nephropathy (presence)	0.18 (0.27)	NE	0.23 (0.27)	NE
Diabetic retinopathy (presence)	0.13 (0.44)	0.23 (0.11)	−0.05 (0.81)	NE
Lp-PLA_2_ (IU/mL)	−0.03 (0.85)	NE	0.34 (0.09)	0.43 (<0.01 **)

CAVI: cardio-ankle vascular index; Lp-PLA_2_: lipoprotein-associated phospholipase A_2_; NE: not extracted; *r*: simple correlation test (Pearson test); β: stepwise multiple linear regression analysis for CAVI. Significance level: * *p* < 0.05, ** *p* < 0.01.
